# Tissue Damage, Not Infection, Triggers Hepatic Unfolded Protein Response in an Experimental Rat Peritonitis Model

**DOI:** 10.3389/fmed.2022.785285

**Published:** 2022-03-16

**Authors:** Andrea Müllebner, Anna Herminghaus, Ingrid Miller, Martina Kames, Andreia Luís, Olaf Picker, Inge Bauer, Andrey V. Kozlov, Johanna Catharina Duvigneau

**Affiliations:** ^1^Ludwig Boltzmann Institute for Traumatology, The Research Center in Cooperation With AUVA, Vienna, Austria; ^2^Department of Biomedical Sciences, Institute for Medical Biochemistry, University of Veterinary Medicine Vienna, Vienna, Austria; ^3^Department of Anesthesiology, University Hospital Düsseldorf, Düsseldorf, Germany

**Keywords:** sepsis, systemic inflammatory response syndrome (SIRS), ER stress, surgical trauma, colon ascendens stent peritonitis (CASP), unfolded protein response

## Abstract

**Background:**

Abdominal surgery is an efficient treatment of intra-abdominal sepsis. Surgical trauma and peritoneal infection lead to the activation of multiple pathological pathways. The liver is particularly susceptible to injury under septic conditions. Liver function is impaired when pathological conditions induce endoplasmic reticulum (ER) stress. ER stress triggers the unfolded protein response (UPR), aiming at restoring ER homeostasis, or inducing cell death. In order to translate basic knowledge on ER function into the clinical setting, we aimed at dissecting the effect of surgery and peritoneal infection on the progression of ER stress/UPR and inflammatory markers in the liver in a clinically relevant experimental animal model.

**Methods:**

Wistar rats underwent laparotomy followed by colon ascendens stent peritonitis (CASP) or surgery (sham) only. Liver damage (aspartate aminotransferase (AST), alanine aminotransferase (ALT) and De Ritis values), inflammatory and UPR markers were assessed in livers at 24, 48, 72, and 96 h postsurgery. Levels of inflammatory (IL-6, TNF-α, iNOS, and HO-1), UPR (XBP1, GRP78, CHOP), and apoptosis (BAX/Bcl-XL) mRNA were determined by qPCR. Splicing of XBP1 (XBP1s) was analyzed by gel electrophoresis, p-eIF2α and GRP78 protein levels using the western blots.

**Results:**

Aspartate aminotransferase levels were elevated 24 h after surgery and thereafter declined with different kinetics in sham and CASP groups. Compared with sham De Ritis ratios were significantly higher in the CASP group, at 48 and 96 h. CASP induced an inflammatory response after 48 h, evidenced by elevated levels of IL-6, TNF-α, iNOS, and HO-1. In contrast, UPR markers XBP1s, p-eIF2α, GRP78, XBP1, and CHOP did not increase in response to infection but paralleled the kinetics of AST and De Ritis ratios. We found that inflammatory markers were predominantly associated with CASP, while UPR markers were associated with surgery. However, in the CASP group, we found a stronger correlation between XBP1s, XBP1 and GRP78 with damage markers, suggesting a synergistic influence of inflammation on UPR in our model.

**Conclusion:**

Our results indicate that independent mechanisms induce ER stress/UPR and the inflammatory response in the liver. While peritoneal infection predominantly triggers inflammatory responses, the conditions associated with organ damage are predominant triggers of the hepatic UPR.

## Introduction

Abdominal surgery is the most efficient treatment of intra-abdominal sepsis. However, surgical trauma and peritoneal infection lead to the activation of multiple stress and inflammatory pathways. An exaggerated response can cause systemic inflammatory response syndrome (SIRS). Despite worldwide efforts to improve treatment and clinical outcomes, the mortality rate of sepsis, septic shock, and the consecutive multiorgan dysfunction syndrome (MODS) in humans remains very high (1). Notably, sepsis-associated liver failure and dysfunction are associated with a poor prognosis (2).

The liver plays a particular role in SIRS, as it mounts the acute phase response and represents the source but also a target organ of inflammatory mediators. The liver is a major regulator of immune and inflammatory responses at the systemic level (3). In response to infection and inflammation, the liver adapts its metabolism and switches protein synthesis toward the acute phase reactants (4). For these tasks, the hepatocytes critically depend on the functional endoplasmic reticulum (ER). However, SIRS is associated with profound derangements of the hepatic metabolism and the capacity to produce proteins. Recently, these derangements have been attributed to a dysfunctional ER of hepatocytes, a condition termed ER stress. ER stress is meanwhile considered an early sign of hepatocyte dysfunction preceding liver dysfunction caused by sepsis and SIRS (5, 6).

Endoplasmic reticulum stress elicits the unfolded protein response (UPR), an adaptive response that aims at restoring cellular protein homeostasis (7). Three ER stress sentinels drive UPR in a concurrent manner. Activation of inositol-requiring protein 1-2 (IRE1α) leads to alternative splicing of X-Box binding protein 1 (XBP1) mRNA, an early indicator for ER stress. Proteolytic cleavage of activating transcription factor 6 (ATF6) releases its cytosolic portion. The spliced isoform of XBP1 (XBP1s) and the cleaved ATF6 are potent transcription factors that promote an increase in protein-folding capacity of the ER by enhancing the expression of ER chaperones, such as the glucose-regulated protein 78 kDa (GRP78), and ER-associated protein degradation (8, 9). Activation of the protein kinase R-like ER kinase (PERK) causes translational attenuation by directly phosphorylating the α-subunit of the eukaryotic translation initiation factor 2 (eIF2α) (10). Prolonged activation of PERK commits the cell to UPR-induced apoptosis that can be initiated by increased CCAAT/enhancer-binding protein homologous protein (CHOP) expression. CHOP favors a proapoptotic phenotype by downregulating antiapoptotic mitochondrial proteins of the B-cell lymphoma family such as B-cell lymphoma-extra large (Bcl-XL) causing increased levels of proapoptotic proteins, such as Bcl2-associated X protein (BAX) (11). Thus, UPR can initiate apoptosis in a mitochondria-dependent manner, if ER stress remains unresolved (12).

In the last decade, ER stress and UPR have been explored as biomarkers and therapeutic targets in many diseases (13). Activation of ER stress and UPR have been associated with the induction of liver failure in several critical care disease models, e.g., endotoxemia (14), traumatic/hemorrhagic shock (THS) (15, 16), and sepsis (17–19). Besides trauma and burns, intra-abdominal infections are a common cause of sepsis, which therefore represent an important clinical problem in abdominal surgery (20). For translation into clinical practice, the impact of diverse factors, such as inflammation or tissue damage on ER stress activation in the peritonitis needs further characterization using appropriate biomedical research models.

The colon ascendens stent peritonitis (CASP) model closely mimics the clinical progression of sepsis after intra-abdominal surgery. This experimental peritonitis model is of high-clinical relevance since it allows controlling the severity of sepsis (21). It consists of two independent insults, first tissue damage because of the surgery and second infection because of the bacterial leakage from the gut (22). However, the impact of surgery on the markers for the hepatic stress response has not been addressed so far.

We applied a self-resolving model of CASP, with moderate peritonitis induction, in order to minimize secondary, inflammation-induced tissue injury and damage, which is a frequent septic complication. We assumed this model would be particularly suitable to dissect the effect of surgery and peritonitis-induced inflammation on the progression of UPR and inflammation markers in the liver. The clarification of a causal association between UPR signaling and onset of SIRS is of translational significance, as it implies new medical approaches for preventing liver dysfunction in the clinical situation.

## Materials and Methods

### Animals

**Figure 1:**
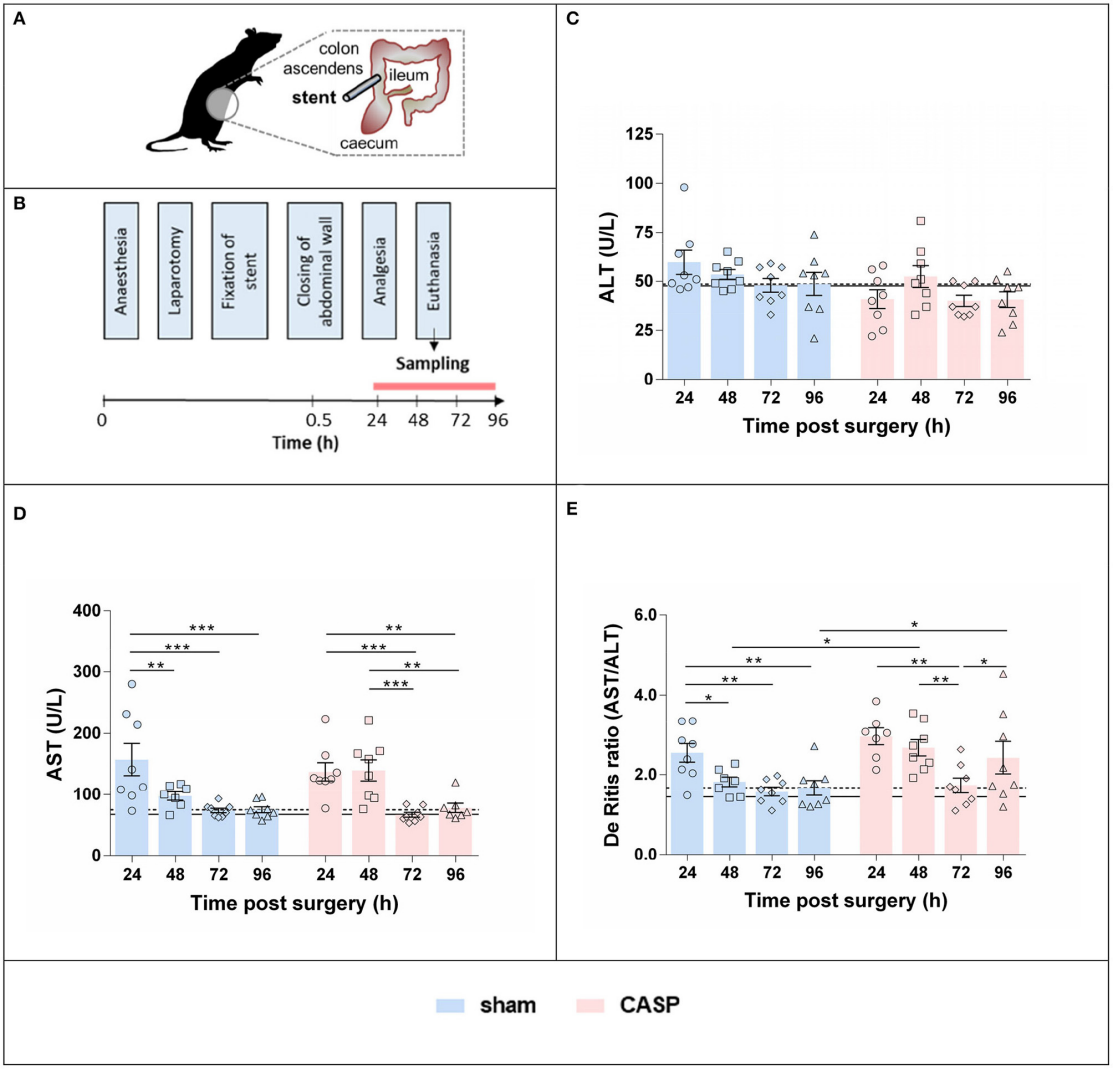
Surgery and peritonitis induce liver damage in a self-resolving model. **(A)** Experimental model for the induction of experimental peritonitis using CASP surgery. **(B)** Plasma and liver samples were collected at consecutive time points after sham and CASP surgery. Plasma levels of **(C)** ALT and **(D)** AST were obtained from a data set determined for a previous study (24) published under Creative Commons-by 4.0 license (25). **(E)** De Ritis ratio was calculated from those data. Data are shown as mean ± SEM per group, with *n* = 8 for all the groups, except for **(D,E)** sham 48 h (*n* = 7), **(D)** CASP 24 h (*n* = 7), and **(D)** CASP 96 h (*n* = 7). The dashed line indicates the mean value of the sham group at 96 h. The solid line indicates the mean value of untreated control animals (*n* = 9). Statistical differences were calculated using two-way ANOVA followed by uncorrected Fisher's LSD test and are indicated by *p < 0.05, **p < 0.01, ***p < 0.001.

In accordance with the ethical guiding principles for animal experiments (23), we aimed at obtaining maximal information from previous animal experiments. We used residual tissue samples of animals investigated in a previous study (24). From animals that did not undergo surgery (untreated control) no more tissue material was available, when we started this study. Because of the limited amount of tissue, we included 8 animals per group, although the previous study comprised 12 animals per group. Animals are described in detail in Herminghaus et al. (24). In brief, liver tissues of 64 adult male Wistar rats (374 ± 23 g body weight) were investigated in this study. Animals were randomly assigned to 8 groups: groups 1–4, sham-operated animals (laparotomy only, 24, 48, 72, and 96 h after surgery) and groups 5–8, CASP with a 14-G stent, 24, 48, 72, and 96 h after surgery (Figure 1A).

### Colon Ascendens Stent Peritonitis/Sham Surgery

Polymicrobial abdominal infection was induced by leakage of feces into the abdominal cavity *via* a stent implanted in the colonic wall (CASP) as previously described (24). This previous study was approved by the local Animal Care and Use Committee (Landesamt für Natur, Umwelt und Verbraucherschutz, Recklinghausen, Germany), and all the experiments were performed in accordance with the NIH guidelines for animal care. In brief, the volatile anesthetic sevoflurane (3.0 Vol%, FiO_2_ 0.5) was used to induce and maintain anesthesia. Buprenorphine was applied at 0.05 mg/kg subcutaneously for analgesia. Animals were laparotomized and a 14-G stent penetrating the colonic wall (ca. 0.5 cm distal to the cecum) was fixed. Sham animals underwent anesthesia and laparotomy as stated earlier, but the stent was fixed outside on the wall of the gut without piercing it. After surgery, animals received analgesia (buprenorphine 0.05 mg/kg in 0.6 ml NaCl subcutaneously every 12 h), but no antibiotics and no additional fluid therapy were applied. The overall survival rate in the sham and CASP group were 100 and 94%, respectively. Animals were euthanized by intraperitoneal injection of pentobarbital (120 mg/kg) 24, 48, 72, or 96 h after sham/CASP surgery. Blood was obtained by cardiac puncture. Livers were collected, aliquots were shock frozen in liquid nitrogen, and stored at −80°C until further processing. The experimental scheme is shown in Figure 1B.

### Plasma Analyses

Plasma levels of alanine aminotransferase (ALT) and aspartate aminotransferase (AST) of all animals included in this study (*n* = 8 per group) and also from untreated control animals (*n* = 9) were taken from a data set determined in a previous study (24) published under Creative Commons-by 4.0 license (25). In brief, plasma was obtained by centrifugation (4°C, 4000 × *g*, 10 min) from blood collected in EDTA tubes and stored at −80°C until further processing. ALT and AST activities were measured in the Central Institute of Clinical Chemistry and Laboratory Medicine of the University Hospital Duesseldorf, Germany (24).

### Gene Expression Analyses

Liver tissue (25–50 mg) was homogenized in 1 ml of TriReagent^®^ (Molecular Research Center Inc., Cincinnati, OH, USA). Total RNA was extracted according to the manufacturer's protocol. Extracted RNA was quantified and purity was checked with an Eppendorf BioPhotometer plus UV/VIS (Eppendorf, Wesseling-Berzdorf, Germany) using absorption at 260 nm and the 260/280 nm ratio, respectively. Reverse transcription of 1 μg of total RNA to cDNA was performed using Superscript^TM^ II reverse transcriptase (200 U/reaction; Invitrogen; Carlsbad, CA, USA) and anchored oligo dT primers (3.5 μmol/l final concentration). Equal aliquots of each cDNA were pooled to generate an internal standard used as a reference for the quantification of qPCR.

Quantitative PCR was performed in reactions of 12 μl containing SYBR^®^ green I (0.5×, Sigma Aldrich, Vienna, Austria), iTaq™ DNA polymerase™ (25 U/L; Bio Rad, Hercules, CA, USA), oligonucleotide primers (250 nmol/l each, Invitrogen; Carlsbad, California, USA), dNTP [200 μmol/l each], and MgCl_2_ (1.5-3 mmol/l). All the reactions were performed in duplicates on a CFX96™ real-time cycler (Bio-Rad, Hercules, California, USA). Details on primer pairs are shown in Supplementary Table S1. Randomly assigned no-reverse transcriptase controls corresponding to ~15% of all the samples investigated, a no-template control, and the internal standard was included in each measurement. ΔCq of no-reverse transcriptase controls to the respective sample was >7 for all the cases, while no-template control never yielded signals.

Data were analyzed using the CFX Manager (version 2.0, Bio-Rad, Hercules, CA, USA) in the linear regression mode. Target gene expression was calculated relative to the internal standard (ΔCq) and normalized by mean ΔCq values of two internal reference genes (hypoxanthine-guanine phosphoribosyltransferase, HPRT, and cyclophilin A, Cyclo), yielding ΔΔCq values, as previously described (26). The ΔΔCq values obtained from the technical replicates were averaged and used for statistical analyses. For visualization, data are presented as fold changes (2^−ΔΔ*Cq*^ values) relative to the mean of the 96 h sham group. The 96 h time point was used as a reference point, owing to the lack of untreated control animals. Our previous study revealed (24) that not only CASP but also the surgical procedure itself transiently affected liver damage markers (AST and ALT) and the mitochondrial function of the liver. However, ALT and AST data obtained from the 96 h sham animals were nearly identical to those of the untreated control animals (Figures 1C–E). Therefore, we assume the values of the 96 h sham animals correspond to physiological levels. This is in line with previously published results (19).

### Western Blot Analyses

Liver tissues were homogenized 1:10 (w/v) in RIPA lysis buffer (25 mmol/l Tris–HCl (pH 8.0), 0.5% Nonidet P-40, 150 mmol/l NaCl, 0.25% sodium deoxycholate, 0.05% sodium dodecyl sulfate (SDS), 1 mmol/l EDTA, and 0.5 mmol/l DTT) freshly supplemented with protease and phosphatase inhibitor cocktails (Roche, Mannheim, Germany). After centrifugation (12,000 × *g*) at 4°C for 10 min, protein concentration in the supernatant was determined using the Bradford method. Western blotting was performed essentially as previously described (15), on specimens of three randomly selected animals per group. Samples (20 μg protein per lane, reduced in Laemmli sample buffer) were separated by sodium dodecyl sulfate-polyacrylamide gel electrophoresis over a separation distance of 7 cm followed by semidry blotting onto nitrocellulose (Hybond ECL; GE Healthcare Life Sciences, Munich, Germany). Blots were first stained with the fluorescent dye ruthenium (II) tris-(bathophenanthroline disulfonate) and overall protein pattern captured on a Typhoon RGB imager (GE Healthcare Life Sciences, Munich, Germany). Immunostaining was performed with specific antibodies against GRP78 (ALX-210-137, Enzo Life Sciences, 1:5,000) or p-eIF2α (No. 9721, Cell Signaling, 1:1000) followed by cross-adsorbed anti-rabbit-HRPO (No. A16104, Life Technologies). Reactive bands were detected by enhanced chemiluminescence (Clarity™ Western ECL Blotting reagent, Bio-Rad) on a Vilber Fusion FX system (Vilber-Lourmat, Eberhardzell, Germany). The overall protein-staining pattern was used as a loading control and for normalization.

### Data Analyses and Statistics

Data were calculated and visualized using GraphPad Prism v6.01 (GraphPad Software Incorporation, La Jolla, California, USA). Outliers were detected by the ROUTs test (Q = 1%) (27) and excluded from analyses. Data were analyzed by two-way ANOVA followed by uncorrected Fisher's LSD test unless otherwise stated. Correlations were calculated using Pearson's correlation coefficient. Differences were considered significant when the *p*-value was < 0.05.

## Results

### Liver Damage Markers Are Increased 24–48 h After Surgery

The values for the plasma levels of liver damage markers, activities of ALT (Figure 1C) and AST (Figure 1D) of untreated control animals (*n* = 9), sham and CASP animals enrolled in this study (*n* = 8 per group), were taken from a previously determined data set (see Materials and Methods, Animals and Plasma Analyses). In the acute phase (24-48 h), AST was elevated two-fold compared to the postacute phase (72–96 h) in both sham and CASP groups. We did not observe any significant difference in AST and ALT levels between sham and CASP groups at any time point (24). In contrast, the De Ritis ratio (AST/ALT) was significantly higher in CASP groups compared to shams at 48 and 96 h (Figure 1E). The 96 h sham group displayed values for ALT and AST, and also the De Ritis ratio, that did not differ significantly from the non-operated control animals (ALT: *p* = 0.9; AST: *p* = 0.3; De Ritis: *p* = 0.4; two-sided, heteroscedastic Student's *t*-test), indicating resolution of liver injury.

### Abdominal Infection Triggered Stress and Inflammatory Response in the Liver 48 h After CASP

**Figure 2:**
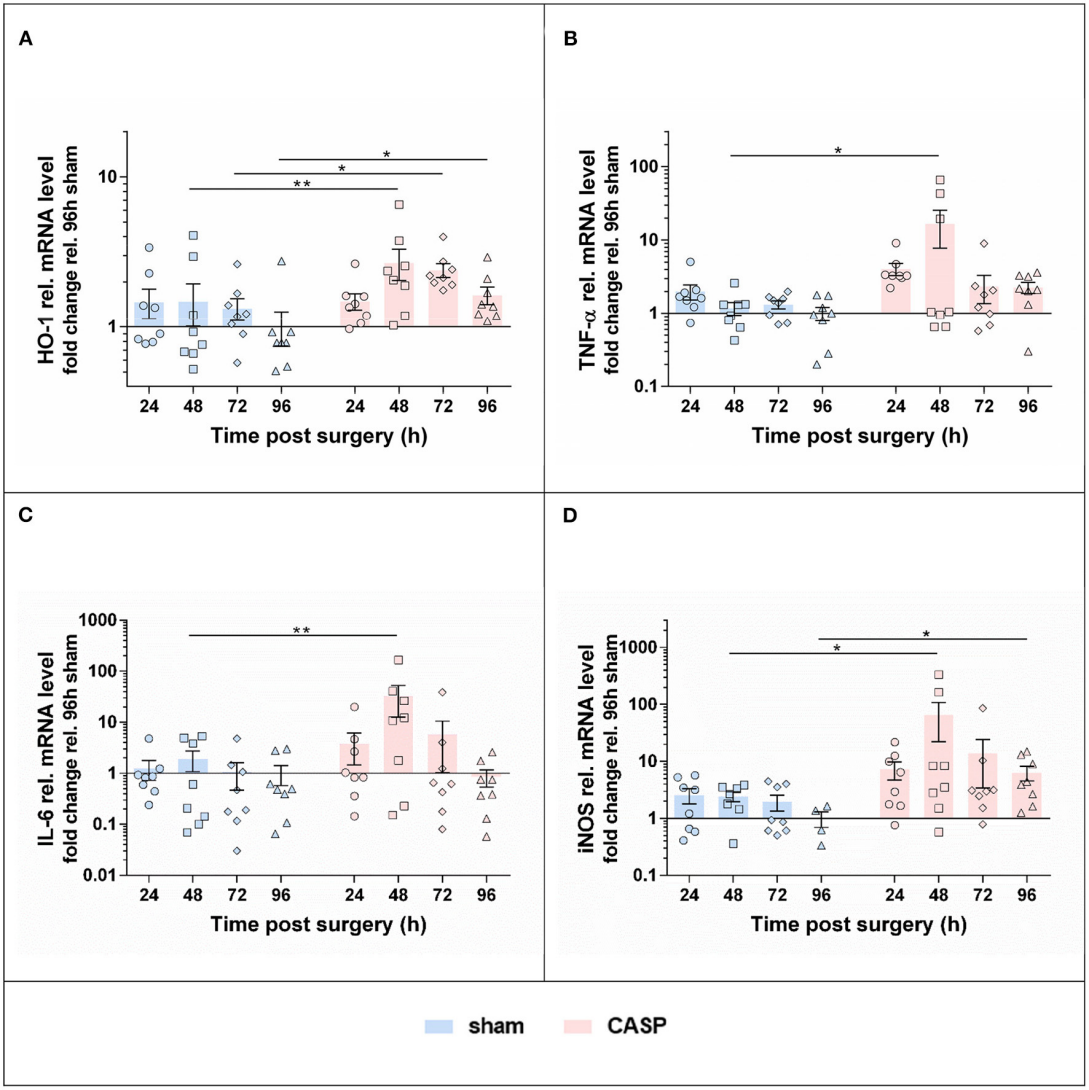
Hepatic inflammatory response induced by abdominal infection peaks at 48 h. Gene expression levels of **(A)** HO-1, **(B)** TNF-α, **(C)** IL-6, and **(D)** iNOS were determined using qPCR in liver samples of rats that underwent sham or CASP surgery. Data are shown as mean ± SEM, with *n* = 8 for all the groups, except for **(D)** sham 48 h (*n* = 7) and **(D)** sham 96 h (*n* = 4). Statistical differences were calculated using two-way ANOVA followed by uncorrected Fisher's LSD test and are indicated by *p < 0.05; **p < 0.01.

To assess the inflammatory response in the liver, we analyzed gene expression levels of key inflammatory markers. The mRNA levels of stress responsive enzyme heme oxygenase 1 (HO-1; Figure 2A) were moderately, albeit, significantly increased in CASP compared to sham-operated animals at 48, 72, and 96 h. The mRNA levels of the proinflammatory cytokines tumor necrosis factor α (TNF-α; Figure 2B) and interleukin 6 (IL-6; Figure 2C), and inducible NO synthase (iNOS; Figure 2D), an enzyme required for bactericidal activity, were significantly higher in CASP compared with sham animals at 48 h after surgery. In addition, iNOS was significantly higher in CASP animals at 96 h (Figure 2D). These data show that our experimental CASP model causes abdominal infection, which is capable to trigger an inflammatory response in the liver.

### XBP1 Splicing and eIF2α Phosphorylation Are Triggered by Surgical Stress

**Figure 3:**
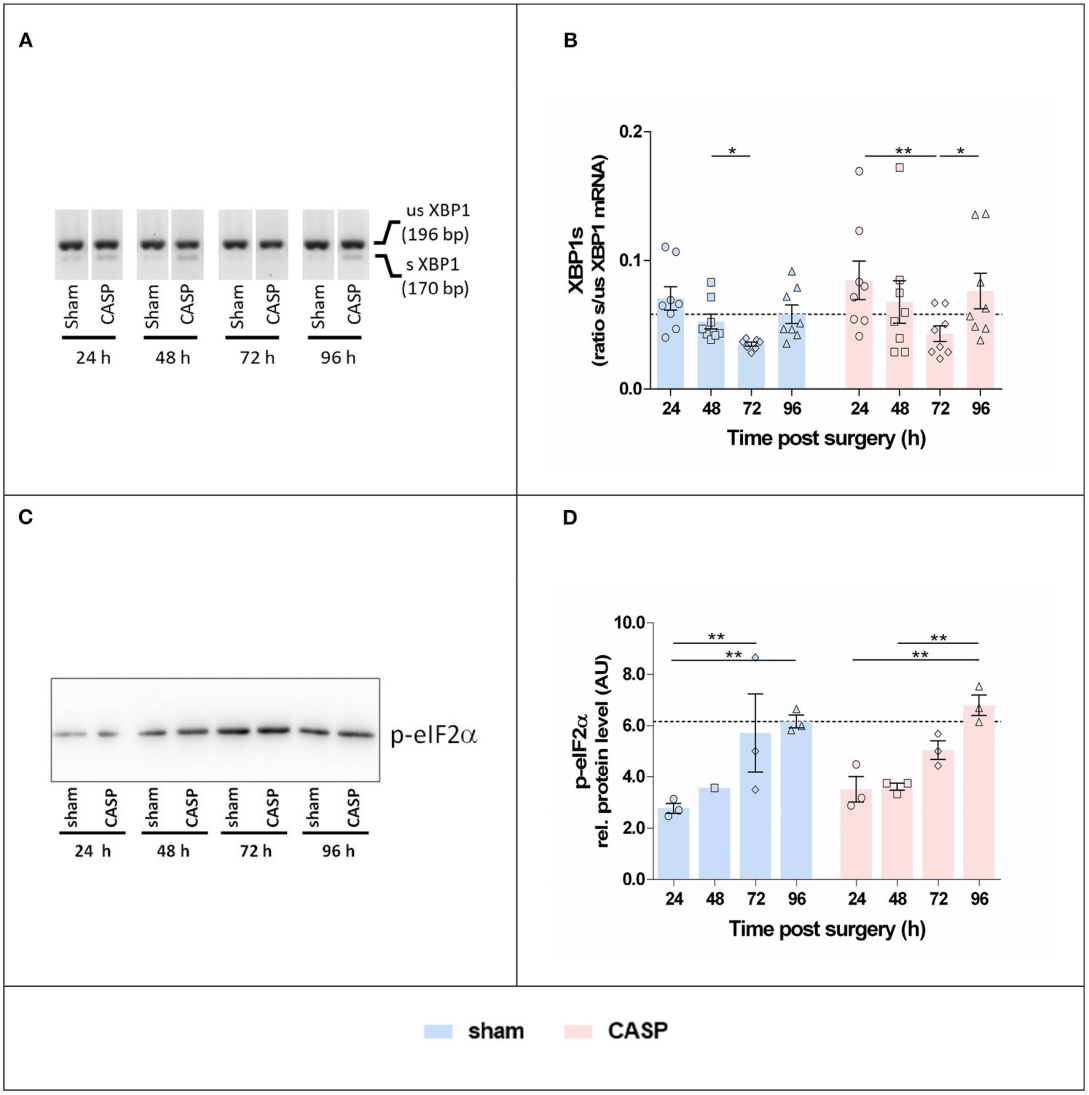
Surgery induces hepatic XBP1 splicing and eIF2α phosphorylation. **(A)** Representative agarose gel electrophoresis of XBP1 PCR products showing the occurrence of the splice variant in the liver. **(B)** XBP1s indicated as the ratio of spliced (s) to unspliced (us) XBP1 mRNA determined by densitometric analyses. Data are given as mean ± SEM (*n* = 8 per group). **(C)** Liver homogenates were analyzed by SDS-PAGE and immunostaining for p-eIF2α. An exemplary blot is shown (the entire blot is shown in Supplementary Figure S1A). **(D)** Band intensities were normalized to the total protein of the respective gel lanes. Values are given as mean AU (arbitrary units) ± SEM (*n* = 3 per group, except for sham 48 h *n* = 1). The dashed line indicates the mean value of the sham group at 96 h. Statistical differences were calculated using two-way ANOVA followed by uncorrected Fisher's LSD test and are indicated by *p < 0.05, **p < 0.01.

We studied the activation of canonical UPR signaling in response to CASP and sham operation by quantifying XBP1s and detecting p-eIF2α. XBP1s levels were the highest at 24 h after surgery and declined until 72 h after surgery. Subsequently XBP1s increased again. Changes reached significance between 48 and 72 h in the sham group and 24, and 72 h and 96 h in CASP animals (Figures 3A,B). The p-eIF2α continuously increased throughout the observation period in sham and CASP-operated animals (Figures 3C,D). Within the sham group, we found significantly higher levels of p-eIF2α at 72 h and 96 h compared with the 24 h time point. The CASP group displayed similar kinetics with significantly higher levels at 96 h compared with the values determined at 24 and 48 h. Although the CASP group displayed higher levels of XBP1s and p-eIF2α at the late time point (96 h), the differences between the sham and CASP group failed to be significant. This suggests that ER stress sentinels, IRE1α and PERK, were activated by surgery-associated stress rather than by moderate peritoneal infection.

### Unfolded Protein Response Is Transient and Peaks at 24 h After Surgery

**Figure 4:**
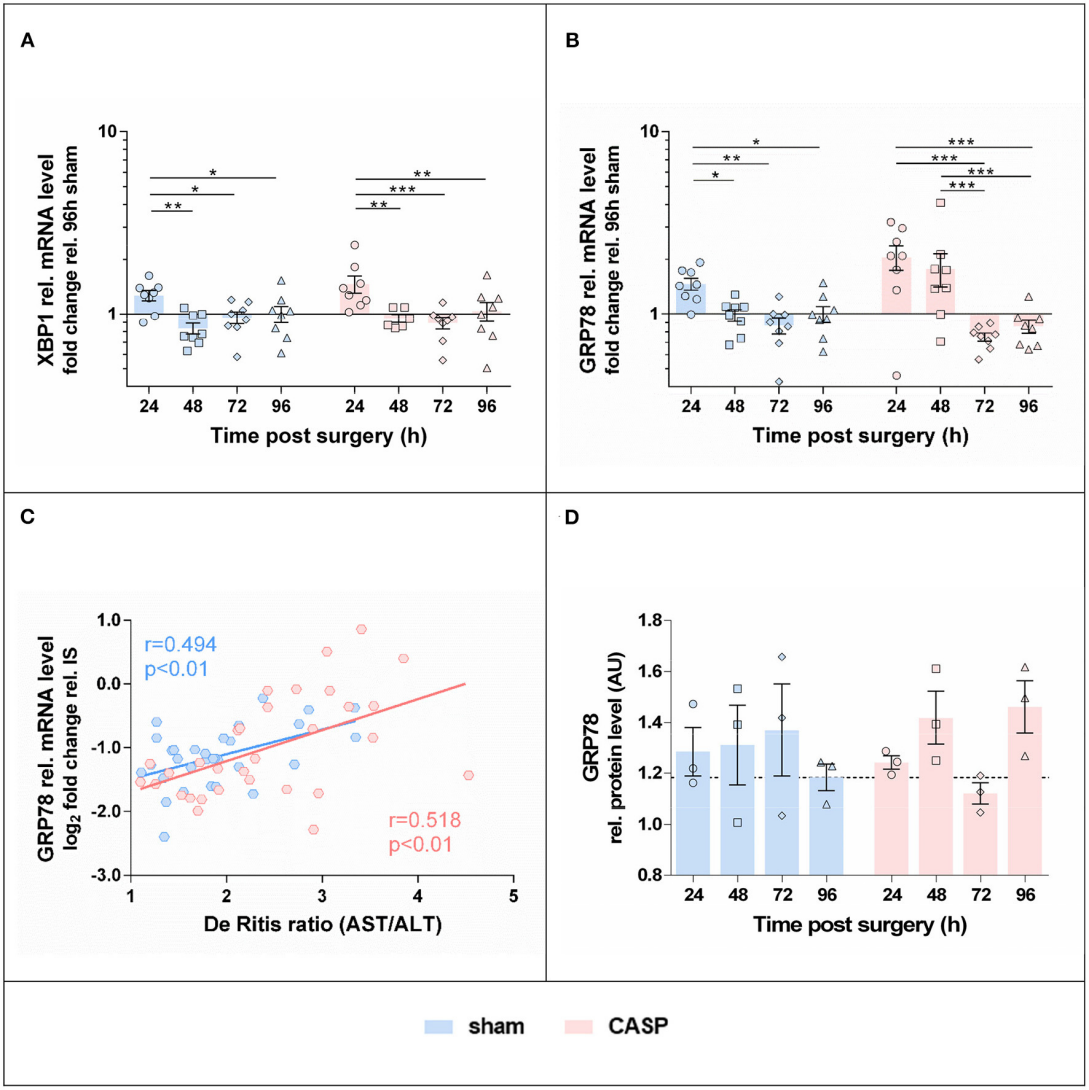
Gene expression of UPR markers in the liver is highest at 24 h after surgery. Gene expression levels of **(A)** XBP1 and **(B)** GRP78 in the liver of sham and CASP operated rats were determined by means of qPCR. Data are shown as mean ± SEM (*n* = 8 per group). **(C)** Pearson correlation between GRP78 mRNA level and De Ritis ratio of sham (*n* = 31) and CASP (*n* = 31) operated animals. **(D)** GRP78 protein abundance in liver homogenates of single animals was analyzed by SDS-PAGE and immunostained for GRP78 (blot is shown in Supplementary Figure S1B). Band intensities of specific staining were normalized to the total protein of the respective gel lanes. Values are given as mean AU (arbitrary units) ± SEM (*n* = 3 per group). The dashed line indicates the mean value of the sham group at 96 h. Statistical differences were calculated using two-way ANOVA followed by uncorrected Fisher's LSD test and are indicated by *p < 0.05; **p < 0.01, and ***p < 0.005.

We next examined gene expression of UPR target genes, XBP1 and GRP78. Protein expression of GRP78 was additionally determined. The highest levels of XBP1 (Figure 4A) and GRP78 mRNA (Figure 4B) were found in sham and CASP groups at 24 h after surgery; however, no differences between the sham and CASP groups were found. In addition, we observed a close correlation of GRP78 mRNA levels with the marker for organ damage De Ritis ratio (Figure 4C) with a correlation coefficient (r) of 0.494 (*p* < 0.01) for the sham group and 0.518 (*p* < 0.01) for the CASP group, respectively. Although differences were not significant there was a trend toward lower GRP78 protein levels in the sham group at 96 h compared with the levels at 24 h, while we observed a slight increase at 96 h for the CASP group (Figure 4D; Supplementary Figure S1).

### Unfolded Protein Response Triggered a Proapoptotic Shift in the Liver 48 h After Surgery

**Figure 5:**
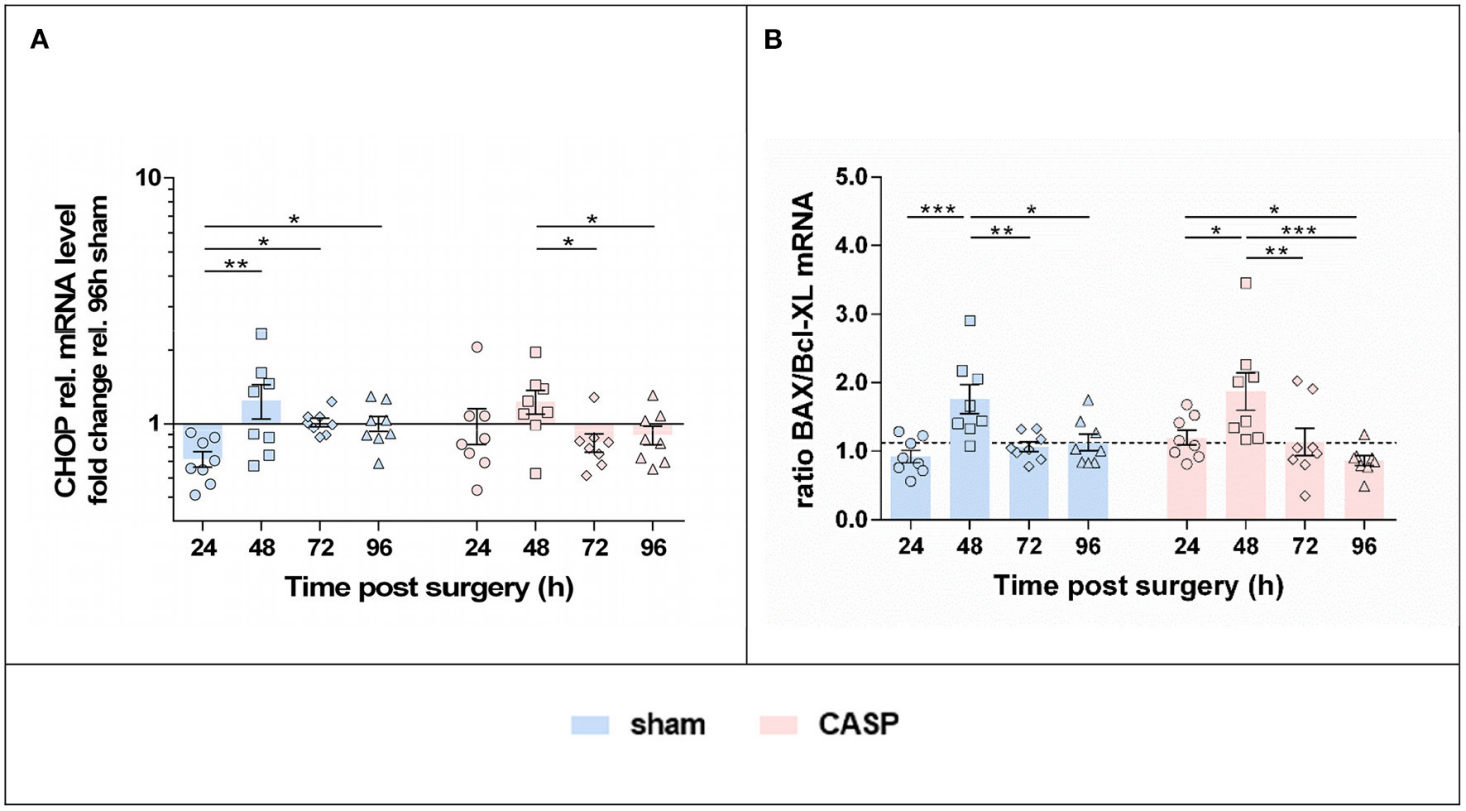
UPR triggers a pro-apoptotic shift with maximum at 48 h after surgery. Gene expression levels of **(A)** CHOP, **(B)** BAX and Bcl-XL mRNA were determined by means of qPCR in liver samples after sham and CASP surgery. Data from BAX and Bcl-XL expression are presented as a ratio. The dashed line indicates the mean value of the sham group at 96 h. All data are shown as mean ± SEM (*n* = 8 per group). Statistical difference was calculated using two-way ANOVA followed by uncorrected Fisher's LSD test and is indicated by *p < 0.05, **p < 0.01, and ***p < 0.005.

Since we found that p-eIF2α levels continued to increase after surgery, which is a sign for sustained activation of the PERK axis of the UPR, we next analyzed markers indicative of apoptosis activation. In both groups, we found the highest levels of CHOP, a downstream target of PERK activation, at 48 h after surgery. Thereafter, CHOP gene expression levels declined (Figure 5A). In addition, markers of the mitochondria-triggered apoptotic pathway, involving BAX and Bcl-XL, displayed a transient proapoptotic shift in both groups (Figure 5B). Compared with all the other time points, the ratio of the proapoptotic BAX to the antiapoptotic Bcl-XL mRNA was significantly increased at 48 h after surgery in both, CASP and sham animals. However, no differences were found between the sham and CASP groups.

### Unfolded Protein Response Activation Is Associated With the Surgical Stress

Considering that the degree of tissue damage caused by the surgical procedures was similar in all the experimental animals, the elicited effects are supposed to be influenced mainly by the time passed after surgery. In contrast, effects elicited by the peritoneal infection should distinguish sham animals from the CASP animals. In order to test the hypothesis that tissue damage, not peritoneal infection acts as a direct trigger for the hepatic UPR, we analyzed our data for both main effects, “time after surgery” and “peritoneal infection” and in addition for a potential interaction of both the conditions. We observed that inflammatory markers (TNF-α, IL-6, iNOS, and HO-1) were exclusively associated with the peritoneal infection, while UPR markers (XBPs, p-eIF2α, XBP1, GRP78, and CHOP) exclusively associated surgical stress (Table 1). There was no remarkable interaction between peritoneal infection and surgery for most markers. However, the interaction found for GRP78 mRNA in our study indicates that infectious stress in the peritoneum is capable of modulating the altitude of hepatic GRP78 gene expression that was triggered by the surgical stress.

**Table 1:** Main effects of peritoneal infection and surgical stress on inflammatory markers, unfolded protein response, and interaction of both the conditions determined by two-way ANOVA.

**Variable**		**Main effect (** ***p*** **-value)**		**Interaction (*p*-value)**
		**Peritoneal infection**	**Surgery**	
AST	Activity	0.638	**<0.0001**	0.133
ALT	Activity	**0.008**	0.157	0.289
De Ritis ratio	(AST/ALT)	**0.002**	**0.0002**	0.396
TNF-α	mRNA	**0.002**	0.132	0.516
IL-6	mRNA	**0.035**	0.112	0.233
iNOS	mRNA	**0.0004**	0.4690	0.872
HO-1	mRNA	**0.000**	0.141	0.314
p-eIF2α	Protein	0.733	**0.0008**	0.697
XBP1s	mRNA	0.074	**0.007**	0.975
GRP78	mRNA	0.202	**<0.0001**	**0.044**
XBP1	mRNA	0.404	**<0.0001**	0.625
GRP78	Protein	0,765	0.680	0.134
CHOP	mRNA	0.897	**0.006**	0.118
BAX/Bcl-XL	mRNA	0.995	**<0.0001**	0.164

*Significant effects (p < 0.05) are indicated by bold values*.

### Activation of UPR Is Directly Correlated With the Level of Liver Damage During Abdominal Infection

**Figure 6:**
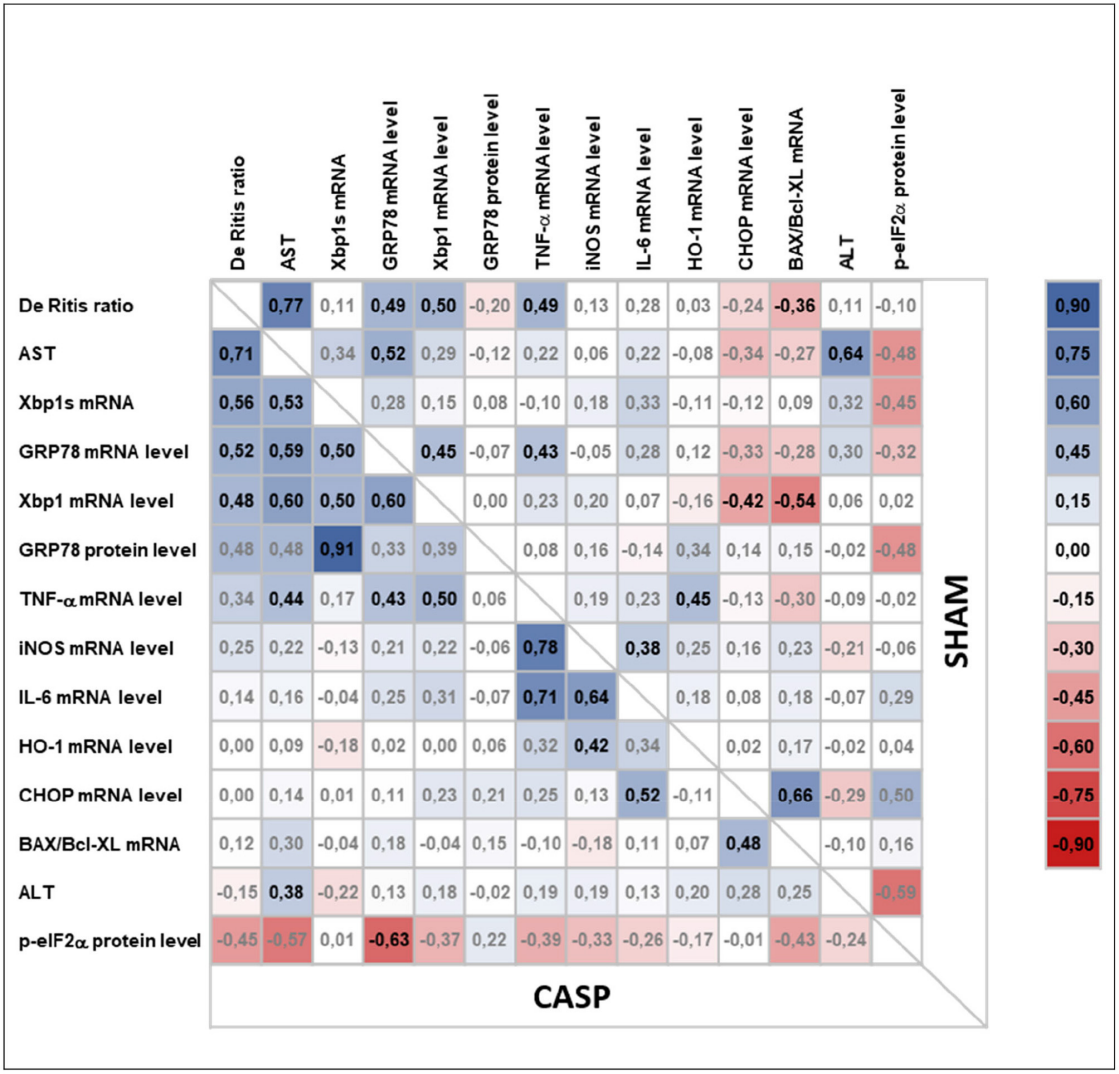
Pearson correlation analysis of liver damage markers, UPR, and inflammatory response in animals subjected to surgery (sham) or CASP. Markers for organ damage (plasma ALT and AST and De Ritis ratio) and levels of hepatic markers for UPR (XBP1s mRNA, XBP1 mRNA, GRP78 mRNA/protein, p-eIF2α protein, CHOP mRNA), inflammatory response (IL-6 mRNA, TNF-α mRNA, iNOS mRNA), general stress response (HO-1 mRNA), as well as BAX/Bcl-XL mRNA ratio as a marker for a proapoptotic phenotype were correlated with each other in both, CASP and sham animals, using Pearson correlation (*n* = 4–8 for all mRNA data, *n* = 1–3 for all the protein data). In the upper right and the lower left part correlation coefficients (*r*) calculated from sham and CASP animals, respectively, are shown. A correlation plot was prepared using Microsoft Excel 2016. Positive correlations are highlighted in blue, negative correlations in red. Significant correlations are indicated by bold letters.

We next analyzed the correlations among organ damage, UPR, or inflammation markers within sham and CASP animals using Pearson correlation (Figure 6). We found that UPR target genes GRP78 and XBP1 correlated significantly with liver damage markers De Ritis ratio (GRP78: sham *r* = 0.49, *p* < 0.01 and CASP *r* = 0.52, *p* < 0.01; XBP1: sham *r* = 0.50, *p* < 0.01 and CASP *r* = 0.48, *p* < 0.01) and AST (GRP78: sham *r* = 0.52, *p* < 0.01 and CASP *r* = 0.59, *p* < 0.01; XBP1: CASP *r* = 0.60, *p* < 0.01) in animals of both groups (Figure 6). Interestingly, these correlations were stronger among each other in animals of the CASP group. In addition, a strong positive correlation of XBP1s with liver damage markers (De Ritis ratio: *r* = 0.56, *p* < 0.01; AST: *r* = 0.53, *p* < 0.01) and with GRP78 (*r* = 0.91, *p* < 0.01) protein expression was found in CASP animals. Moreover, only in the CASP animals, the gene expression of the inflammatory marker TNF-α correlated with XBP1 mRNA (*r* = 0.5, *p* < 0.01). In sham animals, we found inverse correlations of CHOP and BAX/Bcl-XL with XBP1 mRNA (CHOP: *r* = −0.42, *p* < 0.05; BAX/Bcl-XL: *r* = −0.54, *p* < 0.01), which were not present in CASP animals. In contrast, no correlations between liver damage and markers of the inflammatory response were found. Taken together, our data suggest that not peritoneal infection, but organ damage triggers hepatic UPR.

### Infectious Stimulants Are Weak, but Organ Damage-Triggering Factors Are Strong Inducers of UPR in the Liver

Since an upregulated hepatic UPR has been shown in several inflammatory animal models, including our own (15, 28), the question arises, which condition acts as a predominant trigger; the inflammation-inducing stimuli, or the tissue damage, which is accompanying severe inflammatory processes. To address this question more profoundly, we determined clustering of representative markers by reanalyzing data sets from two different acute experimental models, a THS model and an endotoxic shock model (*i.v*. application of LPS), which were part of studies previously published (15, 28). While the THS model induces initially acute tissue damage, *via* ischemia/reperfusion injury, intravenous LPS application leads acutely to a fulminant inflammatory response. Thus, analyzing a very early time point (2 h) in both models, we expected to see the predominantly triggered responses, without superimposing secondary effects. The applied analytical approach, of ranking the normalized effects of investigated markers (refer to Methods to Supplementary Figure S2 in Supplementary Material), allows a direct comparison of marker clustering in both models. We found significant differences between the THS (2 h after trauma and hemorrhage) and the endotoxic shock group (LPS, 2 h). The LPS group showed higher cumulative ranks of inflammatory markers (iNOS, TNF-α) with lower ALT ranks, and lower ranks for UPR (CHOP, XBP1, XBP1s, and GRP78) compared with the THS animals. In contrast, the THS group showed higher ranks of ER-stress markers (CHOP, XBP1, and XBP1s) and higher ALT ranks, while an association with the inflammatory markers was nearly absent (Supplementary Figure S2). Of note: HO-1 levels were significantly increased compared with controls in both models, supporting the observation of HO-1 as an exquisite marker of the general hepatic cell stress.

In addition, our experiments performed with an immortalized liver cell line (BRL3A) described in the Supplementary Material (Methods to Supplementary Figure S3), support the finding of the weak capacity of inflammatory mediators to directly induce hepatic UPR. Although the BRL3A cells are capable of exquisitely responding to ER stress inducers, such as tunicamycin and thapsigargin (Supplementary Figure S3A), incubation with inflammatory mediators raised the expression of IL-6, without clearly affecting the UPR response markers, GRP78 and CHOP (Supplementary Figure S3B).

These additional data support the assumption that not infectious stimuli, but the conditions associated with organ damage are the predominant triggers of the hepatic UPR seen in our CASP model.

## Discussion

### Animal Model

Abdominal infections are an important clinical problem. In Germany, abdominal infections cause 28.7% of all sepsis cases (29). Primary peritonitis caused by diverticulitis or Morbus Crohn, or secondary peritonitis through anastomotic failure after surgical procedures is a frequent problem in the clinical setting.

The CASP model is a well-established and clinically relevant animal model of polymicrobial sepsis. The stent used in CASP leads to continuous leakage of feces into the abdomen, and therefore, closely mimics the clinical course of diffuse peritonitis in patients with steadily increasing systemic infection and inflammation (22). The severity of sepsis and resulting mortality can be controlled in CASP models, as it is directly depending on the size of the stent (21). Mortality occurs already at early time points in the experimental models of severe sepsis, such as CASP with large stent, or cecal ligation and puncture models (22). In the model of moderate peritonitis, which was applied in this study, the survival rate was more than 90% until 96 h after surgery (24). Therefore, this model is characterized as non-lethal but self-resolving peritonitis. Using this model, we previously demonstrated that CASP transiently compromised liver mitochondria early (24–48 h) after surgery (24). The transient nature of CASP-induced effects on functional parameters of the liver confirms the moderate and self-resolving character of the present peritonitis model.

### Organ/Cell Damage

The levels of the liver damage markers AST, as published previously (24), and the De Ritis ratio were only moderately elevated. AST and De Ritis ratio were significantly higher at the early time point (24 h) in response to the surgical stress. Thereafter, these values declined successively supporting the self-resolving character of the CASP model. Abdominal infection only slightly modulated AST values, which resulted in moderately, albeit significantly higher De Ritis ratios in the CASP groups at 48 and 96 h after surgery. Thus, the surgical procedure exerted an acute, but transient stress that was associated with a moderate liver-damaging potential. Moderate peritonitis contributed little to liver damage, which occurred predominantly in response to surgery. We assume that this reaction was the consequence of tissue damage related to the surgery. The increased expression of ER stress response genes in the blood cells of patients 1 day after cardiac surgery with cardiopulmonary bypass supports our assumption (30).

### Hepatic Inflammatory Response

In contrast to organ damage markers, expression of markers of inflammation in liver tissues showed a strong response to and a clear association with the abdominal infection. In CASP compared with the sham animals, gene expression of proinflammatory markers was maximally and significantly increased at 48 h. Thereafter, gene expression levels declined with HO-1 and iNOS still being significantly higher in the sham group up to the latest time point investigated (96 h). We attribute this late effect to the continuously increasing systemic response to infection and inflammation induced by CASP, as shown before (22). These data show that surgery contributes little if anything to the inflammatory response in the liver, which is essentially triggered by the abdominal infection. Of note, we have determined the inflammatory markers at gene expression levels, which reflect a quick response to infection. Thus, it can be assumed that in the present model, abdominal infection induced by CASP takes about 2 days (48 h) to reach the maximum. This is also the time point, at which high-mortality rates can be observed in severe sepsis models and septic patients (21, 31, 32). We assumed that UPR would follow the same kinetics if triggered by infectious stimuli.

### Hepatic Unfolded Protein Response

Contrary to this assumption, the kinetics of expression of ER stress and UPR-related markers were different from those of the proinflammatory markers. The changes observed for the UPR-related markers were moderate, but most strikingly, markers associated with IRE1α activation were maximal at 24 h after surgery. Furthermore, we could not determine a significant effect of CASP on the UPR-related markers investigated.

Unfolded protein response activation in consequence of tissue damage (33), particularly because of hypoxia or ischemia/reperfusion (15, 16), has been demonstrated in the last couple of years. Thus, we assume that hepatic ER stress and UPR are a consequence of circulating damage-associated signals (possibly danger-associated molecular patterns) rather than inflammatory mediators.

This association was addressed more profoundly by reanalyzing data sets from two different acute models, a THS model and an endotoxic shock model (*i.v*. application of lipopolysaccharide (LPS)), which were part of studies previously published (15, 28). Both experimental models are associated with a substantial loss of animals (up to 50%) and are, in contrast to the CASP model, characterized by a nearly immediate response of the liver (15, 28). Therefore, we considered a very early time point (2 h) most suitable to dissect the direct impact of induced tissue damage vs. induced inflammation on the manifestation of the hepatic ER stress response.

The infectious stimulus, LPS, triggered predominantly an increased inflammatory response that was initially not associated with substantial organ damage (28) and only a weakly upregulated hepatic UPR at this time point. In contrast, THS, which triggered significant organ damage (15), was associated with a strongly upregulated hepatic UPR, while an inflammatory response was absent at this early time point. Of importance, in the THS model XBP1 and XBP1s nearly instantly followed organ damage as indicated by the increased levels of ALT (15). These data indicate that conditions associated with organ damage rather than infectious stimuli operate as direct triggers of the hepatic UPR. This assumption is further supported by our additional experiments using cultured immortalized liver cells, in which we show that inflammatory mediators were capable to induce an inflammatory response, but not a substantial ER stress response.

We found that UPR, which was triggered primarily by tissue damage, is associated with IRE1α and PERK activation. IRE1α activation, through the expression of XBP1s, has been extensively associated with cell survival (34). In the CASP model, IRE1α was temporarily activated early (24 h) after surgery, while the PERK-eIF2α-p-eIF2α pathway was activated throughout the entire observation period. Phosphorylation of eIF2α inhibits translation initiation resulting in a reduction of protein load in the ER, except for transcripts related to ER stress resolution (35). Persistent PERK-ATF4-CHOP signaling can commit the cell to apoptosis (36). Indeed, CHOP levels peaked 48 h after surgery and resulted in transiently higher BAX/Bcl-XL ratios indicating a proapoptotic switch at the consecutive time point (48 h) in the present model. In addition, eIF2α phosphorylation and CHOP activation are associated with metabolic dysregulation during hepatic ER stress (37).

Although individual UPR-related targets increased only moderately in this model, the entity of upregulated UPR-related markers following tissue trauma because of the surgery likely reflects an early effort to rescue tissue function upon danger signaling, as was previously suggested (38).

In addition, both the XBP1 and the downstream target GRP78 correlated significantly with AST and De Ritis ratio in the present CASP model. Although a correlation does not imply a causal relationship, it is noteworthy that organ damage markers did not correlate with inflammatory markers, but with ER stress markers. Pharmacological induction of ER stress has been shown to result in increased mortality after trauma (33). In contrast, inhibition of ER stress has been shown to protect livers against ischemia/reperfusion injury in a model of hepatectomy (39). This indicates that possibly danger-associated molecular patterns, released in substantial amounts during trauma or surgery, but also secondary to substantial systemic inflammatory conditions are triggers of liver cell death employing mechanisms that involve ER stress pathways.

### Interaction of Hepatic UPR With CASP-Induced Inflammatory Response Pathways

Even though abdominal infection had no significant effect on the expression of UPR markers, we found a stronger association between XBP1s and GRP78 with liver damage markers in rats that underwent CASP. Furthermore, CASP altered the kinetics of GRP78 gene expression following surgical trauma toward a later decline, suggesting cooperation between inflammatory and ER stress pathways. To the best of our knowledge, no experiments could show a direct induction of UPR by inflammatory mediators, such as cytokines. However, the secondary organ damage, which is typically accompanying inflammatory conditions, could well explain the increased ER stress response seen in SIRS. Vice versa, ER stress is capable to trigger inflammatory pathways acting as synergizing components in several pathologies (40). Both UPR branches, IRE1α and PERK can directly activate nuclear factor kappa B (NF-κB) (41, 42) leading to the production of inflammatory cytokines. ER stress activating IRE1α is further linked to TNF-α-mediated cell death through the adaptor protein tumor necrosis factor α receptor-associated factor 2 (TRAF2) and NF-κB (43). Therefore, cell death signals synergize in response to inflammation triggered by TNF-α, which also employs NF-κB. TNF-α levels in the plasma of CASP animals were elevated 96 h after surgery in the present model (24). The increased De Ritis ratio in the CASP group at this time point possibly reflects a converged synergism of cell death pathways. Although the low mortality of this CASP model indicates that the elicited inflammatory response may be self-resolving, it was sufficient to transiently affect mitochondrial function in the liver (24). Interestingly, mitochondrial damage driven by caspase 2 has been described as a mechanism underlying hepatocyte death upon ER stress that operated *via* NLRP3 inflammasome activation (44, 45).

Given the good outcome of our experimental rats, which were young and showed no clinical signs when enrolled in the study, we assume that the damage triggered hepatic UPR was well balanced and exerted a beneficial role in the present CASP model. However, since ER stress is a critical inflammation triggering factor, the role of surgery modulating UPR warrants closer consideration in patients suffering from comorbidities, i.e., metabolic diseases (46). Based on our results, we suggest therapeutic approaches, which target the ER to maintain liver function in conditions associated with inflammatory processes, such as sepsis. Of note, certain anesthetics or antibiotics have been shown to modulate ER stress induction in the liver (47–50). Thus, the choice of an appropriate anesthetic protocol during surgery followed by a suitable antibiotic therapy might help to shape UPR and limit consecutive liver damage.

### Limitations

This study was focused on the progression of ER stress/UPR markers exclusively in the liver as a remote target organ in the peritonitis and abdominal surgery, because of its central role for the system. However, we did not analyze the local effects of peritonitis. Several studies have already highlighted the relevance of intestinal ER stress in peritonitis that is tightly linked to deranged intestinal tissue homeostasis and immunity (51, 52), and particularly the impairment of the intestinal barrier function (53). Further studies will be necessary to clarify the source or the nature of the compounds that trigger the ER stress/UPR and inflammatory response in the liver.

## Conclusion

Using a clinically relevant experimental sepsis model, we found that surgical trauma activates hepatic UPR, presumably because of tissue injury. UPR activation occurred early and preceded the inflammatory response in the liver. This indicates that hepatic UPR and the inflammatory response are triggered by different mechanisms. Our data further suggest that secondary tissue injury, as it occurs in septic complications, may influence the severity of ER stress and UPR-mediated liver cell dysfunction. Thus, the hepatic ER appears to be an important target for shaping UPR in order to prevent liver dysfunction in abdominal surgery and severe SIRS.

## Data Availability Statement

The original contributions presented in the study are included in the article/Supplementary Material, further inquiries can be directed to the corresponding author.

## Ethics Statement

The animal study was reviewed and approved by Landesamt für Natur, Umwelt und Verbraucherschutz, Recklinghausen, Germany (experiments were performed in accordance with the NIH Guidelines for Animal Care).

## Author Contributions

AM wrote the first draft of the manuscript, performed the statistical analysis and the graphical data presentation. AH and OP performed and supervised animal experiments. IM performed and interpreted WB analysis. AM and MK performed and interpreted PCR analysis. AL provided critical feedback and revised the manuscript. JD, IB, and AK designed and supervised the study. JD and AK provided critical feedback and wrote the final version of the manuscript. All authors gave intellectual input and approved the final version of the manuscript.

## Funding

AM was supported by the Austrian Research Promotion Agency with a Ph.D. Grant: Industrienahe Dissertation (849090). MK was supported by an Internship for female students from the Austrian Research Promotion Agency (FFG). AL received funding from the European Union's Horizon 2020 Research and Innovation Program under the Marie Skłodowska-Curie Grant Agreement No. 675448.

## Conflict of Interest

The authors declare that the research was conducted in the absence of any commercial or financial relationships that could be construed as a potential conflict of interest.

## Publisher's Note

All claims expressed in this article are solely those of the authors and do not necessarily represent those of their affiliated organizations, or those of the publisher, the editors and the reviewers. Any product that may be evaluated in this article, or claim that may be made by its manufacturer, is not guaranteed or endorsed by the publisher.

## Abbreviations

ATF4: activating transcription factor 4
ATF6: activating transcription factor 6
BAX: Bcl2 associated X protein
Bcl: B-cell lymphoma
Bcl-XL: Bcl-extra large
BRL3A: buffalo rat liver 3A cell line
CASP: colon ascendens stent peritonitis
CHOP: CCAAT/enhancer-binding protein homologous protein
Cyclo: cyclophilin A
eIF2α: α-subunit of the eukaryotic translation initiation factor 2
ER: endoplasmic reticulum
GRP78: glucose regulated protein 78 kDa
HO-1: heme oxygenase 1
HPRT: hypoxanthine-guanine phosphoribosyltransferase
iNOS: inducible NO synthase
IL-6: interleukin 6
IRE1α: inositol-requiring enzyme-1
LPS: lipopolysaccharide
MODS: multi-organ dysfunction syndrome
NF-κB: Nuclear factor kappa B
PERK: protein kinase R-like endoplasmic reticulum kinase
p-eIF2α: phosphorylated α-subunit of the eukaryotic translation initiation factor 2
SIRS: systemic inflammatory response syndrome
TNF-α: tumor necrosis factor α
THS: traumatic/hemorrhagic shock
TRAF2: tumor necrosis factor α receptor-associated factor 2
UPR: unfolded protein response
XBP1: X-Box binding protein 1
XBP1s: spliced isoform of XBP1.
