# Supplementary material for: Tissue Damage, Not Infection, Triggers Hepatic Unfolded Protein Response in an Experimental Rat Peritonitis Model
Source: Front Med (Lausanne). 2022 Mar 16;9:785285. doi: 10.3389/fmed.2022.785285 (PMC8965740; doi:10.3389/fmed.2022.785285)
Supplement: Supplementary file 1 [file Data_Sheet_1.docx]

# Tissue damage, not infection, triggers hepatic unfolded protein response in an experimental rat peritonitis model

Supplementary Material

**Supplementary Table 1:** Sequence and source of primer pairs used in this study.

| **Target** | **Accession number** | **Primer sequences** | **Position on plus strand** | **Product Length** | **Source** |
| --- | --- | --- | --- | --- | --- |
| **Cyclo** | M19533 | TAT CTG CAC TGC CAA GAC TGA GTG | 381 | 127 | (1) |
|  |  | CTT CTT GCT GGT CTT GCC ATT CC | 507 |  |  |
| **HPRT** | NM_012583 | CTC ATG GAC TGA TTA TGG ACA GGA C | 179 | 123 | (2) |
|  |  | GCA GGT CAG CAA AGA ACT TAT AGC C | 301 |  |  |
| **TNF-α** | X66539 | TGC CTC AGC CTC TTC TCA TT | 94 | 376 | (1) |
|  |  | TGT GGG TGA GGA GCA CAT AG | 469 |  |  |
| **IL-6** | NM_012589.1 | CCG GAG AGG AGA CTT CAC AG | 154 | 161 | (3) |
|  |  | ACA GTG CAT CAT CGC TGT TC | 314 |  |  |
| **iNOS** | NM_012611.3 | AGG CAA GCC CTC ACC TAC TT | 2654 | 161 | (3) |
|  |  | GTG GGG TTG TTG CTG AAC TT | 2814 |  |  |
| **HO-1** | NM_012580.2 | CCA GCC ACA CAG CAC TAC | 441 | 293 | (1) |
|  |  | GCG GTC TTA GCC TCT TCT G | 733 |  |  |
| **GRP78** | NM_013083.2 | GTT CTG CTT GAT GTG TGT CC | 1424 | 349 | (1) |
|  |  | TTT GGT CAT TGG TGA TGG TG | 1772 |  |  |
| **XBP1** | NM_001004210.1 | GAG TCC AAG GGG AAT GGA GT | 435 | 196 | (4) |
|  |  | ACA GGG TCC AAC TTG TCC AG | 630 |  |  |
| **CHOP** | NM_024134.2 | TTG GGG GCA CCT ATA TCT CA | 219 | 263 | (1) |
|  |  | CTC CTT CAT GCG CTG TTT CC | 481 |  |  |
| **BAX** | NM_017059.2 | AAA GTG CCC GAG CTG ATC A | 471 | 153 | (5) |
|  |  | AGC CAC AAA GAT GGT CAC TGT CT | 623 |  |  |
| **Bcl-XL** | NM_001033672.1 | AAT GAA CTC TTT CGG GAT GGG | 717 | 126 | (5) |
|  |  | CCA ACT TGC AAT CCG ACT CA | 842 |  |  |

| A  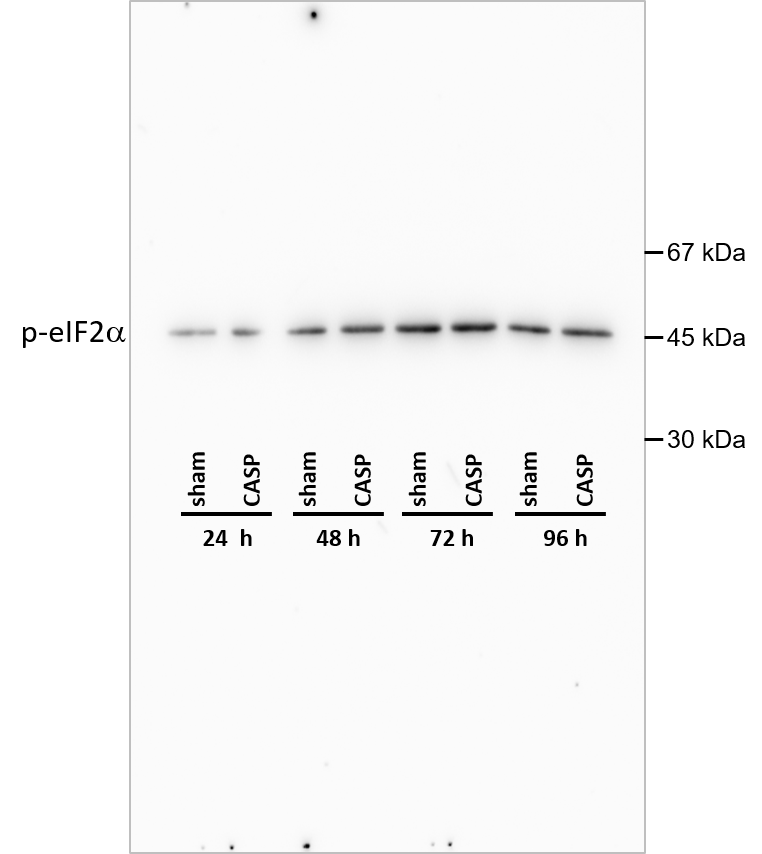 | B  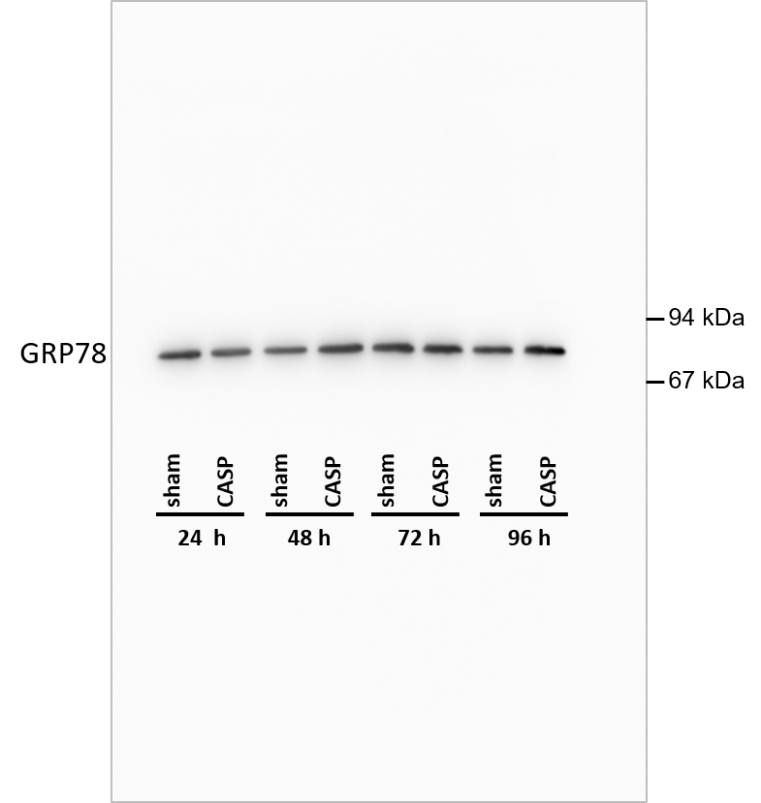 |
| --- | --- |
| **Supplementary Figure 1:** p-eIF2α and GRP78 protein abundance in liver homogenates after sham or CASP surgery. Liver homogenates were analyzed by SDS-PAGE and immunostained for p‑eIF2α or GRP78. Exemplary whole blots of p-eIF2α (A) and GRP78 (B) staining are shown. | |

| 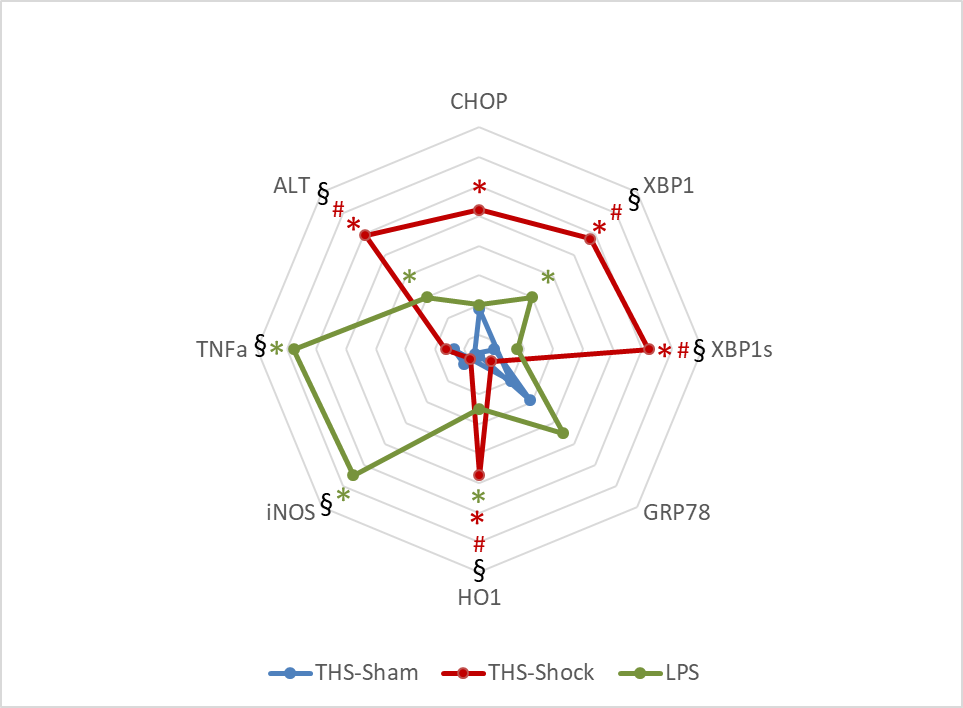 |
| --- |
| **Supplementary Figure 2:** **Hepatic UPR is associated primarily with organ damage, rather than with induction of inflammatory response.** Radar chart showing mean ranks of normalized values from rats subjected to an inflammatory stimulus (LPS *i.v.* 2 h, n=7, green line) or tissue damage (traumatic/hemorrhagic shock, THS-Shock, 2 h after onset of hemorrhage, n=8, red line), and the respective sham control (THS-Sham, n=8, blue line) for visualizing predominating type of response. Values were calculated from data sets of previous studies, in which experimental details can be found (5,6). Values for plasma levels of organ damage marker (ALT) and mRNA expression levels of ER stress (CHOP, GRP78, XBP1, XBP1s), inflammatory (iNOS, TNF-α) and general cell stress (HO-1) response markers were normalized for allowing comparison and ranking as is described beneath in the methods to supplementary figure 2. Higher mean group ranks indicate higher differences to the untreated control animals (n=7/8). For indicating significant differences to the respective control groups normalized data were used and Kruskal Wallis, followed by Dunn test was performed (*, p<0.05, relative to untreated control; #, p<0.05, relative to THS-Sham). Differences between THS and LPS group were calculated with ranked values using non-parametric Wilcoxon test (IBM SPSS Statistics 27), and are indicated (§, p<0.05 significant differences between). |

## Methods to supplementary figure 2:

## Association of UPR and inflammatory response with liver damage and induction of inflammatory response in comparable *in-vivo* models

Two data sets from previously published studies (5,6) were reanalysed. Data were obtained from a study using an endotoxic shock model, 2 h after injection of LPS (6 mg/kg, *i.v.*; n = 6/7) reflecting immediate response to an infectious/inflammatory stimulus. Details regarding the groups’ description and the experimental approach can be found elsewhere (6). The second data set was taken from a study using an experimental model of traumatic/hemorrhagic shock (THS) consisting of surgery and severe haemorrhage followed by inadequate volume resuscitation corresponding to a time point of 2h after onset of haemorrhage and includes THS (n=8) and sham (n=8) animals. Details regarding the groups’ description and the experimental approach can be found elsewhere (5). In both studies a group of corresponding untreated control animals were included for comparison and levels for organ damage (plasma ALT), as well as hepatic ER stress response (CHOP, GRP78, XBP1 and XBP1s mRNA), inflammatory response (iNOS, TNF-α mRNA) and general cell stress response (HO-1 mRNA) levels were recorded.

For allowing direct comparison, the two data sets were normalized, using respective untreated control values as reference. From this group the median was calculated and all data were expressed relative to the median of the reference animals. To calculate differences between groups normalized data were used and Kruskal-Wallis, followed by Dunn test (GraphPad Prism v6.01; GraphPad Software, Inc., La Jolla, CA, USA) was applied. To enable visualization of the elicited effects independently of the magnitude, the normalized values of the experimental animals (THS, sham, LPS) were ranked in increasing order according to their effect size and mean group ranks were calculated. Statistical differences between mean ranks of LPS and THS-Shock group were calculated using non-parametric Wilcoxon test (IBM SPSS Statistics 27; IBM, New York, USA).

| **A**   | **B**  **** |
| --- | --- |
| **Supplementary Figure 3:** **Effect of ER stressors and inflammatory mediators (IM) on expression of ER stress markers in immortalized rat liver cells.** BRL3A cells were incubated with (A) classical ER stressors or (B) conditioned medium (CM), which was generated using white blood cells treated without (Ctrl.-CM) or with LPS to elicit production of inflammatory mediators (IM-CM) as described beneath in the methods to supplementary figure 3. Levels of mRNA were calculated relative to vehicle (A) or untreated (B) controls. Data are shown as mean +/-SEM (n=2/3). Levels of UPR marker mRNA (GRP78, CHOP, XBP1) are presented on primary, IL‑6 mRNA levels on the secondary Y-axis in B. Significant differences were calculated relative to vehicle (A) or untreated (B) control (Student’s t-test, paired, 2 sided; *, p<0.05; **, p<0.01; ***, p<0.005). | |

## Methods to supplementary figure 3:

## Cell culture

Adherently growing cells from buffalo rat liver 3A cell line (BRL3A, ECACC) were cultivated in Coon’s F-12 medium with 5% fetal calf serum (PAA). For the experiments, cells were grown in six-well plates until reaching a confluency of 70–80%. BRL3A cells were incubated with different concentrations of thapsigargin (1.6, 8, 40 nmol/L), tunicamycin (8, 40, 200 ng/L) or vehicle (dimethyl sulfoxide) for 6 h and for 3 h or 6 h with RPMI-1640 medium (untreated control), control conditioned medium or inflammatory mediator containing conditioned medium. Thereafter, RNA was extracted, reverse transcribed and used for qPCR analyses, as described in Materials and Methods, Gene Expression Analyses.

## Preparation of conditioned media

Medium containing inflammatory mediators was generated as described elsewhere (3). Briefly, conditioned medium was prepared from isolated rat white blood cells set at a density of 1 × 10^6^ cells/mL. Cells were incubated for 24 h at 37°C with RPMI-1640 medium alone (Ctr.-CM) or supplemented with LPS (E. coli Serotype 026:B6, 6 μg/ml; IM-CM) to obtain control or inflammatory mediators containing medium, respectively. The cell suspension was centrifuged (10 min, 400×g, 4°C), the supernatants harvested and stored at −80°C until being used. Inflammatory cytokines were determined by Myriad RBMTM (Austin, TX, USA), and were exclusively present in the inflammatory mediators containing medium (IFNγ, 4.6 pg/mL; IL-1α, 290 pg/mL; TNF-α, 0.6 pg/mL; MCP1, 1335 pg/mL).

**References:**

1. Kozlov A V., Catharina Duvigneau J, Hyatt TC, Raju R, Behling T, Hartl RT, Staniek K, Miller I, Gregor W, Redl H, et al. Effect of estrogen on mitochondrial function and intracellular stress markers in rat liver and kidney following trauma-hemorrhagic shock and prolonged hypotension. *Mol Med* (2010) **16**:254–261. doi:10.2119/molmed.2009.00184

2. Müllebner A, Moldzio R, Redl H, Kozlov A V., Duvigneau JC. Heme degradation by heme oxygenase protects mitochondria but induces ER stress via formed bilirubin. *Biomolecules* (2015) **5**:679–701. doi:10.3390/biom5020679

3. Weidinger A, Dungel P, Perlinger M, Singer K, Ghebes C, Duvigneau JC, Müllebner A, Schäfer U, Redl H, Kozlov A V. Experimental data suggesting that inflammation mediated rat liver mitochondrial dysfunction results from secondary hypoxia rather than from direct effects of inflammatory mediators. *Front Physiol* (2013) **4 JUN**:138. doi:10.3389/fphys.2013.00138

4. Müllebner A, Moldzio R, Redl H, Kozlov AV, Duvigneau JC. Heme degradation by heme oxygenase protects mitochondria but induces ER stress via formed bilirubin. *Biomolecules* (2015) **5**: doi:10.3390/biom5020679

5. Duvigneau JC, Kozlov A V., Zifko C, Postl A, Hartl RT, Miller I, Gille L, Staniek K, Moldzio R, Gregor W, et al. Reperfusion does not induce oxidative stress but sustained endoplasmic reticulum stress in livers of rats subjected to traumatic-hemorrhagic shock. *Shock* (2010) **33**:289–298. doi:10.1097/SHK.0b013e3181aef322

6. Nürnberger S, Miller I, Catharina Duvigneau J, Kavanagh ET, Gupta S, Hartl RT, Hori O, Gesslbauer B, Samali A, Kungl A, et al. Impairment of endoplasmic reticulum in liver as an early consequence of the systemic inflammatory response in rats. *Am J Physiol - Gastrointest Liver Physiol* (2012) **303**:1373–1383. doi:10.1152/ajpgi.00056.2012
